# Phase I/II study of cisplatin combined with weekly paclitaxel in patients with advanced non-small-cell lung cancer

**DOI:** 10.1038/sj.bjc.6601672

**Published:** 2004-02-24

**Authors:** N Yoshimura, S Kudoh, T Mukohara, S Yamauchi, M Yamada, T Kawaguchi, Y Nakaoka, K Hirata, J Yoshikawa

**Affiliations:** 1 Department of Respiratory Medicine, Graduate School of Medicine, Osaka City University, 1-4-3, Asahi-machi, Abeno-ku, Osaka 545-8585, Japan; 2 Internal Medicine, Osaka City Sumiyoshi Hospital, 1-2-16, Higasikagaya, Suminoe-ku, Osaka 559-0012, Japan; 3 Internal Medicine, Osaka City Kita Hospital, 5-4-8, Nishikujyou, Konohana-ku, Osaka 554-0012, Japan; 4 Osaka Japan Railway Hospital, 1-2-22, Matsuzaki-cyou, Abeno-ku, Osaka 545-0053, Japan

**Keywords:** weekly paclitaxel, cisplatin, non-small-cell lung cancer (NSCLC)

## Abstract

To determine the maximum-tolerated dose (MTD) and the recommended dose (RD) of paclitaxel administered weekly with a fixed dose of cisplatin, and to assess the toxicity and activity of this combination, we conducted a phase I/II trial in patients with advanced non-small-cell lung cancer (NSCLC). In this study, patients with stage IIIB/IV NSCLC were eligible. Paclitaxel, at a starting dose of 40 mg m^−2^ week^−1^ on days 1, 8, and 15, was combined with a fixed dose of cisplatin 80 mg m^−2^ on day 1. Chemotherapy was given in a 4-week cycle. In this phase I/II study, 38 patients were enrolled. Dose-limiting toxicities (DLT) were neutropenia, fatigue, and omission of treatment due to leucopenia, thrombocytopenia, or febrile neutropenia. The MTD and RD were estimated to be 70 mg m^−2^. Of the 37 assessable patients, 23 had a partial response and one had a complete response. Overall response rate was 62.1% (95% confidence interval (CI): 46.5–77.7%). The progression-free survival, the median survival time, and the 1-year survival rate were 5.5 months, 13.7 months, and 56.9%, respectively. This regimen is tolerable and very active against advanced NSCLC, and its efficacy should be confirmed in a phase III study.

Lung cancer is a leading cause of cancer death in many industrialised countries, with a 5-year survival rate of only 14% (Wingo *et al*, 1995). Non-small-cell lung cancer (NSCLC) accounts for approximately 75% of all lung cancer, and surgery offers the best chance of cure and long-term survival. Unfortunately, the majority of patients present with disease not amenable to surgery because it is either locally advanced or has metastasised. Chemotherapy for advanced NSCLC is often considered ineffective or excessively toxic. However, meta-analyses have demonstrated that chemotherapy results in a small improvement in survival and quality of life for patients compared with supportive care alone (Grilli *et al*, 1993; Marino *et al*, 1994).

During the past decade, several drugs with novel mechanisms of action and significant activity against NSCLC have been identified, including paclitaxel, docetaxel, vinorelbine, gemcitabine, and irinotecan (Lilenbaum and Green, 1993). Combination of one or two of these agents with a platinum compound has yielded in high response rate and prolonged survival (Langer *et al*, 1995; Crino *et al*, 1997; Abratt *et al*, 1998; Le Chevalier *et al*, 1998; Sandler *et al*, 2000). The Eastern Cooperative Oncology Group (ECOG) conducted a randomised study to determine whether any of three chemotherapy regimens was superior to cisplatin and paclitaxel for patients with advanced NSCLC (ECOG 1594) (Schiller *et al*, 2002). A total of 1207 patients with advanced NCSLC were randomly assigned to a reference regimen of cisplatin and paclitaxel or to one of three experimental regimens: cisplatin and gemcitabine, cisplatin and docetaxel, or carboplatin and paclitaxel. Neither response rate nor survival differed significantly between patients assigned to receive cisplatin and paclitaxel and those assigned to receive any of the three experimental regimens. None of the four chemotherapy regimens offered a significant advantage over the others in the treatment of advanced NSCLC.

Steps such as weekly administration of taxan, nonplatinum doublet chemotherapy, triplet chemotherapy, and use of molecular targeted agents are called for. Among these, weekly administration of paclitaxel has been introduced to chemotherapy for advanced NSCLC and several other types of tumours. In *vitro* experiments and clinical trials have suggested that prolonged exposure to paclitaxel, through either continuous infusion schedules or weekly administration, can lead to enhanced cytotoxicity with maintenance of a favourable toxicity profile (Liebmann *et al*, 1993; Georgiadis *et al*, 1997; Zhan *et al*, 1997). In an attempt to increase drug exposure time, weekly schedules of intravenous paclitaxel were initiated and exhibited promising activity and manageable toxicity for several types of tumours. Akerley *et al*(2003) conducted a phase II trial for patients with chemotherapy-naive, advanced-stage NSCLC. Paclitaxel, 150 mg m^−2^, was administered over 3 h during weeks 1–6 of an 8-week cycle. In total, 38 patients were treated. Grades 3–4 granulocytopenia occurred in 39% of patients. There were no deaths due to toxicity. Grade 2 or 3 neuropathy occurred in 29 and 24% of patients, respectively. There were 16 partial responses (42%). The median survival period was 12.3 months, and the 1-year and 2-year survival rates were 52 and 26%, respectively. Seidman *et al*(1998) conducted a clinical trial in patients with metastatic breast cancer who had received prior therapy. A total of 30 patients received sustained weekly paclitaxel therapy at an initial dose of 100 mg m^−2^. Grade 3/4 neutropenia occurred in four patients, but febrile neutropenia was not observed. Peripheral neuropathy prohibited dose escalation above 100 mg m^−2^, and grade 3 neuropathy was observed in two of 21 patients at ⩽100 mg m^−2^.

Cisplatin is still the most active drug for NSCLC and is efficacious when combined with paclitaxel. We therefore investigated the combination of a weekly paclitaxel and cisplatin regimen.

## PATIENTS AND METHODS

### Patient selection

In the phase I study, patients were enrolled if they met the following criteria: (1) age ⩽75 years, (2) histological or cytological diagnosis of NSCLC; (3) unresectable stage IIIB or IV disease; (4) performance status (PS) of 0, 1, or 2 on the ECOG scale with a predicted life expectancy of at least 12 weeks; (5) measurable or evaluable disease, (6) no prior or only one regimen of chemotherapy; (7) any previous chemotherapy or radiation therapy had been completed more than 4 weeks before enrollment and patients had recovered from any adverse effects; (8) adequate major organ function as documented by a WBC count ⩾4000 *μ*l^−1^, platelet count ⩾100 000 *μ*l^−1^, haemoglobin ⩾9.5 g dl^−1^; total serum bilirubin ⩽1.5 mg dl^−1^, AST and ALT ⩽2 × the institutional upper limit of normal, serum creatinine ⩽1.1 mg dl^−1^, PaO_2_ ⩾70 Torr, and normal ECG. Written informed consent was obtained from all patients. Patients were not eligible for study enrollment in any of the following cases: (1) recent (within the past 3 months) myocardial infarction, uncontrolled angina pectoris, or arrythmia, (2) uncontrolled hypertension or diabetes, (3) active infection, (4) pulmonary fibrosis, (5) massive pleural effusion or ascites, or (6) cerebrovascular disease. In the phase II study, patients were not eligible if they had previously received chemotherapy. Other inclusion and exclusion criteria were the same as for the phase I study.

### Treatment plan

The starting dose of paclitaxel was 40 mg m^−2^ week^−1^ administered intravenously (i.v.) on days 1, 8, and 15, increasing 10 mg m^−2^ week^−1^ by steps. Paclitaxel was infused i.v. in 250 ml normal saline over 60 min. Cisplatin was administered along with a programe of forced diuresis that included at least 2000 ml of fluids after paclitaxel infusion over 60 min on day 1. The cisplatin dose was fixed at 80 mg m^−2^. Chemotherapy was given in an every 4-week cycle and repeated over more than two courses until disease progression or unacceptable toxicity occurred. Patients were premedicated with dexamethasone 20 mg and ranitidine 50 mg i.v. and were given diphenhydramine 50 mg orally 30 min before paclitaxel to prevent hypersensitivity reactions. Prophylactic use of recombinant granulocyte colony-stimulating factor (rhG-CSF) was not allowed in the first course and was discouraged during subsequent courses of treatment. Paclitaxel was withdrawn if the WBC count was less than 2000 *μ*l^−1^ and/or the platelet count was less than 70 000 *μ*l^−1^ on day 8 or 15. Subsequent courses of chemotherapy were initiated when WBC count ⩾4000 *μ*l^−1^ and platelet count was ⩾100 000 *μ*l^−1^ after day 29. If WBC counts or platelet counts had not returned to these levels on day 1 of the next course of chemotherapy, both drugs were withheld until full recovery.

### Dose escalation and definition of the maximum-tolerated dose (MTD) and dose-limiting toxicities (DLT)

Three patients for each cohort were evaluated, and sequential dose levels were studied in the absence of DLT during the first treatment cycle. If one or two of the three patients at any dose level experienced DLT, three additional patients were added at that level before escalation. There was no dose escalation for individual patients. The MTD of the combination was defined as the dose level below that which produced DLT in more than one-third of treated patients. Toxicities were graded using the National Cancer Institute common toxicity criteria, version 2.0. Dose-limiting toxicities was defined as (1) febrile neutropenia (fever ⩾38°C with ⩾grade 3 neutropenia), (2) grade 4 neutropenia (⩾4 days) despite receiving rhG-CSF, (3) grade 4 thrombocytopenia, (4) any other grade 3 or 4 nonhaematologic toxicity (except nausea, vomiting, or alopecia), (5) failure to recover from toxicities sufficiently to begin a second course of treatment by day 43, and omission of chemotherapy on day 8 and/or 15 because of toxities. MTD and RD were determined from the toxicity during the first cycle of treatment.

### Dose modifications

In phase I, doses were not reduced or escalated in individual patients. In phase II, subsequent doses were modified on the basis of haematological and nonhaemotological toxicities. If toxicities were observed during the previous cycle, toxicities were observed, the dose of pacitaxel was reduced by 10 mg m^−2^. Toxicity was defined as DLTs in the phase I study ((1) febrile neutropenia (fever ⩾38°C with ⩾grade 3 neutropenia), (2) grade 4 neutropenia (⩾4 days) despite receiving rhG-CSF, (3) grade 4 thrombocytopenia, (4) any other grade 3 or 4 nonhaematologic toxicity (except nausea, vomiting, or alopecia), (5) omission of chemotherapy on day 8 and/or 15 because of toxicity).

### Patient evaluation

Patients were evaluated to determine clinical stage by complete medical history and physical examination, routine chest radiography, bone scintiscan, and computerised tomography of the head, chest, and abdomen. Before the first course, each patient was subject to complete blood count (CBC), including differential count and platelet count, and serum chemistry was used to check renal and hepatic functions, electrolytes and urinalysis. CBC, serum chemistry, electrolytes, urinalysis, and chest radiographs were assessed at least once a week after the initial evaluation. Other appropriate investigations were repeated biweekly or every 4 weeks to evaluate the sites of marker lesions. After completion of chemotherapy, each patient was restaged with all the tests used during the initial work-up.

Tumour response was assessed according to the World Health Organization criteria (WHO, 1979). Tumours were reassessed during treatment with the same imaging method used to obtain baseline tumour measurement. Whenever possible, patients with evidence of tumour response were to have confirmation within 4–6 weeks after initial documentation of response. In addition, time to response, duration of response, time to tumour progression, and survival were determined. External radiology review was performed for all patients.

### Statistics

The 95% confidence intervals (CIs) for estimated response rate were calculated using the binomial distribution. Time-to-event probability curves and the probability of survival at 1 year were estimated using the Kaplan–Meier methods.

## RESULT

### Patient characteristics

In the phase I study, a total of 18 patients were enrolled between July 2000 and February 2001 (Table 1

**Table 1:** Patients characteristics for phase I/II

**Characteristics**	**Phase I**	**Phase II**	**Total**
Patients entered	18	20	38
Age (years)			
Median	63	67	67
Range	47–73	53–74	47–74
Gender			
Male	10	16	26
Female	8	4	12
PS			
0	3	0	3
1	13	19	32
2	2	1	3
Stage			
IIIB	3	4	7
IV	15	16	31
Histology			
Adenocarcinoma	12	10	22
Squamous cell carcinoma	6	9	15
Large cell carcinoma	0	1	1
Prior therapy			
Chemotherapy	4	0	4
Chemoradiotherapy	3	0	3
Radiotherapy	0	2	2
Surgery	2	4	6
None	9	14	23

). A total of 18 were assessable for toxicity. There were 10 men and eight women with a median age of 63 years (range, 47–73). A total of 15 patients had stage IV and three had stage IIIB disease. Adenocarcinoma was the most common histology (*n*=12), followed by squamous cell carcinoma (*n*=6). Four patients had received prior chemotherapy. Three of these patients had received a combination of cisplatin and docetaxel, and one had received vinorelbin alone. Three patients had received prior chemoradiotherapy. Two of them had received weekly carboplatin+irinotecan and concurrent radiotherapy, and the other had received daily carboplatin and concurrent radiotherapy.

In the phase II study, a total of 20 patients were enrolled between April 2001 and December 2001 (Table 1). In total, 20patients were assessable for toxicity and efficacy. There were 16 men and four women with a median age of 67 years (range, 53–74). A total of 16 patients had stage IV and four had stage IIIB disease. None of the patients had received prior chemotherapy.

### Toxicities

For the phase I study, haematologic toxicities and nonhaematologic toxicities are listed in Table 2

**Table 2:** Toxicity for phase I

	**Level 1 (*n=*3)**				**Level 2 (*n=*6)**				**Level 3 (*n=*3)**				**Level 4 (*n=*6)**			
	**Grade**				**Grade**				**Grade**				**Grade**			
**Toxicity**	**1**	**2**	**3**	**4**	**1**	**2**	**3**	**4**	**1**	**2**	**3**	**4**	**1**	**2**	**3**	**4**
Leucocytopenia	—	—	—	—	—	—	—	—	—	—	—	—	—	—	—	—
Neutropenia	—	—	—	—	1	2	2	1	1	2	—	—	—	1	1	2
Febrile neutropenia	—	—	—	—	—	—	—	—	—	—	1	—	—	—	—	—
Thrombocytopenia	—	—	—	—	2	—	—	—	—	—	—	—	—	1	—	—
Anaemia	—	—	—	—	—	—	—	—	—	—	—	—	—	—	—	—
Nausea	3	—	—	—	3	—	—	—	2	—	1	—	1	3	—	—
Vomiting	—	—	—	—	3	—	—	—	—	—	—	—	1	1	—	—
Anorexia	3	—	—	—	5	—	—	—	1	—	—	—	2	3	—	—
Fatigue	—	—	—	—	—	—	—	—	—	—	—	—	2	—	1	—
Neuropathy	—	—	—	—	—	—	—	—	—	—	—	—	1	—	—	—
Alopecia	—	—	—	—	2	—	—	—	—	1	—	—	3	—	—	—
Elevated AST/ALT	—	—	—	—	—	2	—	—	1	—	—	—	—	—	—	—
Elevated *γ*-GTP	—	—	—	—	—	1	—	—	1	—	—	—	—	—	—	—
Allergic reaction	—	—	—	—	—	—	—	—	—	—	—	—	—	1	—	—

. At the dose level of 50 mg m^−2^, two patients experienced DLT. One had paclitaxel administration omitted on day 15 due to leucopenia, and the other developed febrile neutropenia. Three additional patients were added and none experienced DLT. At 60 mg m^−2^, one experienced grade 3 nausea. At 70 mg m^−2^, three patients were initially enrolled, and one developed grade 3 fatigue and had paclitaxel administration omitted on day 8 due to thrombocytopenia. Three additional patients were therefore added. Of the three, two experienced DLT. One developed grade 4 neutropenia lasting more than 4 days despite receiving rhG-CSF, and one had paclitaxel administration omitted on day 15 due to leucopenia. This dose level was determined to be the MTD, and the RD of phase II was estimated to be this dose as well.

In the phase I study, there were a total of 45 cycles of treatment in 18 patients. The median number of cycles was two (range, one to four). Six cycles (13%) were delayed by more than 6 days due to treatment toxicity.

In the phase II study, the 20 patients assessable for safety received a total of 47 cycles of therapy. The median number of cycles was two (range, one to five). Six (13%) of the cycles of treatment were delayed by more than 6 days due to treatment toxicity.

For the phase II study, Table 3

**Table 3:** Toxicity in all cycles for phase II

	**Grade 1**		**Grade 2**		**Grade 3**		**Grade 4**	
**Toxicity**	**No.**	**%**	**No.**	**%**	**No.**	**%**	**No.**	**%**
Leucocytopenia	4	20	8	40	5	25	—	
Neutropenia	3	15	5	25	6	30	2	10
Febrile neutropenia	—		—		2	10	—	
Thrombocytopenia	7	35	—		—		—	
Anaemia	5	25	12	60	3	15	—	
Nausea	11	55	4	20	1	5	—	
Vomiting	5	25	3	15	—		—	
Anorexia	14	70	2	10	2	10	—	
Fatigue	5	15	2	10	2	10	—	
Neuropathy	1	5	1	5	—		—	
Alopecia	10	50	9	45	—		—	
*γ*-GTP	—		—		1	5	—	
Allergic reaction	—		—		1	5	—	
Constipation	1	5	1	5	—		—	
Fever up	1	5	—		—		—	
Creatinine	1	5	—		—		—	
Flushing	1	5	—		—		—	
Infection	—		—		3	15	—	
Arrythmia	—		—		1	5	—	
Cerebral infarction	—		—		—		1	5

lists the overall incidence of haematologic and nonhaematologic toxicities for all patients treated in all cycles. Grade 3/4 neutropenia was the most common adverse event and occurred in 40% of the patients. None of the patients had grade 3/4 thrombocytopenia. During treatment, only two patients (10%) had febrile neutropenia, which was defined as ⩾grade 1 fever with ⩾grade 3 neutropenia. Five (25%) patients received rhG-CSF during study treatment. The major nonhaematologic grade 3/4 adverse events occurring in seven (35%) of the patients were infection (three; 15%), fatigue (two; 10%), and anorexia. Grade 1/2 peripheral neuropathy occurred in only two patients (10%). Neither increased creatinine nor ototoxicity was observed in any patient. Fatigue was a cumulative toxicity observed during this trial. Five (25%) of 20 patients in the first course, six (40%) of 15 patients in the second course, four (57%) of seven patients in the third course, three (75%) of seven patients (75%) in the fourth course, and one (100%) of one patient in the fifth had fatigue.

### Efficacy

In the phase I and II studies, 37 patients were evaluable for response. Overall, one complete response and 23 partial responses were recorded, for a 62.1% (95% confidence interval: 46.5–77.7) objective response rate (Table 4

**Table 4:** Response for phase I/II

**Dose level**	***n***	**CR**	**PR**	**SD**	**PD**	**NE**	**ORR (%)**
Phase 1							
Level 1	3(2)	—	1(1)	2(1)	0	0	33.3
Level 2	6(3)	—	4(3)	1(0)	0	1(0)	67.8
Level 3	3(1)	—	2(1)	0	1(0)	0	67.8
Level 4	6(1)	—	5(0)	0	1(1)	0	83.3
Total	18(7)	—	12(5)	3(1)	2(1)	1(0)	70.6

Phase II	20(0)	1(0)	11(0)	6(0)	2(0)	0(0)	55.0

Phase I/II	38(7)	1(0)	23(5)	9(1)	2(1)	1(0)	62.1

(): number of pretreated patients, *n*=number of patients, CR=complete response, PR=partial response, SD=stable disease, PD=progression disease, NE=not evaluable, ORR=objective response rate.

). One patient could not be evaluated for response. She was found to have pathological N3 disease after the prior surgery. Chemotherapy was performed in the adjuvant setting and she did not have any measurable lesions. Five partial responses occurred in the seven patients who had received prior systemic chemotherapy.

**Figure 1:**
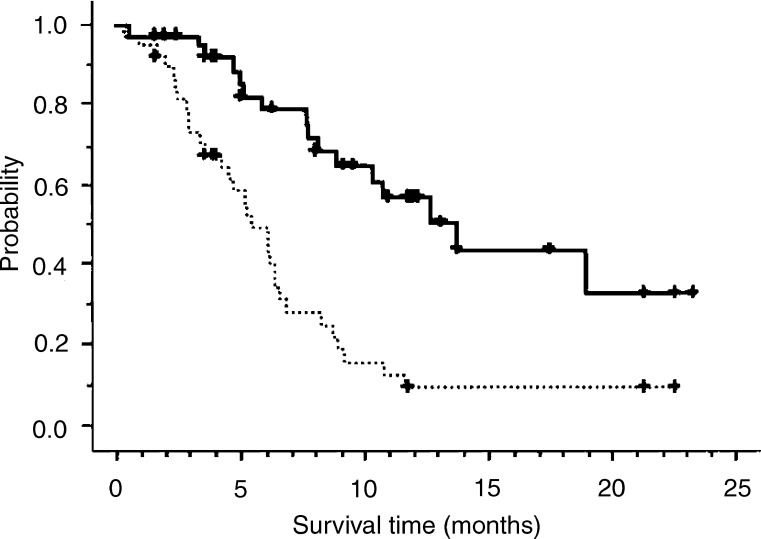
Kaplan–Meier survival curve (—) and time to tumour progression curve (⋯) for the 38 assessable patients are shown. The progression-free survival, median survival time, and 1-year survival rate were 5.5 months, 13.7 months, and 56.9%, respectively. The symbols (+) are for censored patients.

The median time to tumour progression was 5.5 months (range, 0.4–23.2 months), and the median survival was 13.7 months (range, 0.5–23.9 months). The 1-year survival rate was 56.9%. The Kaplan–Meier survival curve and time to tumour progression curve for the 38 assessable patients are shown in Figure 1

.

### Dose-intensity

In the phase I study, the projected dose-intensities of paclitaxel at dose levels 1–4 were 30, 37.5, 45, and 52.5 mg m^−2^ week^−1^. The actual dose-intensities of paclitaxel at each dose level were 29.5(98%), 32.6(87%), 32.1(71%), and 36.7(70%) mg m^−2^ week^−1^. The projected dose-intensity of cisplatin at dose levels 1–4 was 20 mg m^−2^ week^−1^. The actual dose-intensities of cisplatin at each dose level were 19.7(98%), 18.5(93%), 16.5(83%), and 18.7(89%) mg m^−2^ week^−1^.

In the phase II study, the projected dose-intensity of paclitaxel was 52.5 mg m^−2^ week^−1^. The actual dose-intensity of paclitaxel was 41.6 mg m^−2^ week^−1^ (79.2%). The projected dose-intensity of cisplatin was 20.0 mg m^−2^ week^−1^. The actual dose-intensity of cisplatin at each dose level was 19.2 (96.0%) mg m^−2^ week^−1^(Table 5

**Table 5:** Dose intensity for phase I/II

	**PDI (mg m^−2^ week^−1^)**		**ADI (mg m^−2^ week^−1^)**		**RDI (mg m^−2^ week^−1^)**	
	**CDDP**	**PTX**	**CDDP**	**PTX**	**CDDP**	**PTX**
Phase I						
Level 1	20.0	30.0	19.7	29.5	0.98	0.98
Level 2	20.0	37.5	18.5	32.6	0.93	0.87
Level 3	20.0	45.0	16.5	32.1	0.83	0.71
Level 4	20.0	52.5	17.8	36.7	0.89	0.70

Phase II						
	20.0	52.5	19.2	41.6	0.96	0.79

PDI=projected dose intensity; ADI=actual dose intensity; RDI=relative dose intensity; CDDP=cisplatin; PTX=paclitaxel.

).

## DISCUSSION

As did ECOG 1594, other clinical oncology groups such as the Southwestern Oncology Group, Italian Lung Cancer Project, and European Organization for Research and Treatment of Cancer conducted randomised studies to determine whether any new drug plus a platinum compound offered a survival advantage over another reference arm for patients with advanced NSCLC (Kelly *et al*, 2001; Scagliotti *et al*, 2002; Schiller *et al*, 2002; Smit *et al*, 2003). None of the chemotherapy regimens tested offered a significant advantage over the others in the treatment of advanced NSCLC. As a next step in chemotherapy for advanced NSCLC, we investigated a combined weekly paclitaxel and cisplatin regimen. Dose-limiting toxicities were neutropenia, fatigue, and omission of treatment on day 15 due to leucopenia, thrombocytopenia, and febrile neutropenia. The MTD and RD of paclitaxel were estimated to be 70 mg m^−2^. For the 37 assessable patients, the overall response rate was 62.1%. The median survival time and the 1-year survival rate were 13.7 months and 56.9%, respectively. This regimen is thus tolerable and very active against advanced NSCLC.

Carboplatin has been used instead of cisplatin in chemotherapy for NSCLC. Some studies reported that carboplatin combined with weekly paclitaxel was a new and active treatment regimen (Belani, 2001). A phase III study of chemotherapy-naive advanced NSCLC patients was designed to assess whether efficacy in patients receiving a paclitaxel/carboplatin combination was similar to that in patients receiving a paclitaxel/cisplatin combination (Rosell *et al*, 2002). The authors concluded that paclitaxel/cisplatin yielded a response rate similar to and median survival significantly longer than paclitaxel/carboplatin. These results suggested that cisplatin-based chemotherapy should be the first treatment option for NSCLC. We therefore used cisplatin instead of carboplatin in combination with paclitaxel.

Akerley *et al*(2003) conducted a phase II trial for patients with chemotherapy-naive, advanced-stage NSCLC. Paclitaxel, 150 mg m^−2^, was administered over 3 h during weeks 1–6 of an 8-week cycle. A total of 38 patients were treated. Grade 3/4 granulocytopenia occurred in 39% of patients. There were no deaths due to toxicity. Grade 2 or 3 neuropathy occurred in 29 and 24% of patients, respectively. Grade 2/3 anorexia and nausea occurred in 11 and 8% of patients, respectively. In our phase II study, in all cycles grade 3/4 neutropenia was the most common adverse event and occurred in 40% of patients. Neuropathy was mild, at grade 1 or 2 (10%). On the other hand, grade 2/3 anorexia and nausea occurred in four (20%) and five (25%) patients, respectively. Although cisplatin was added in our regimen, the frequency of gastrointestinal toxicity was generally equivalent to that with a weekly paclitaxel regimen. Peripheral neuropathy and allergic reaction were mild compared with those associated with paclitaxel weekly regimen, as a result of the low dose of paclitaxel used in our regimen.

As a standard arm for NSCLC in a phase III study, 175 mg m^−2^ of paclitaxel, administered over a 3-h period on day 1, followed by 80 mg m^−2^ of cisplatin on day 1 every 21 days, is used (Giaccone, 2002). Grade 4 neutropenia was observed in 8.8% of patients treated with this regimen and 10% of patients treated with our regimen. Grade 3 febrile neutropenia was observed in 1.9% of patients treated with that regimen and 10% of patients treated with our regimen. Grade 3 sensory neuropathy was observed in 2.5% of patients treated with that regimen, while grade 3 neuropathy was seen in no patients treated with our regimen. Grade 3 nausea was observed in 6.3% of patients treated with that regimen and 5% of patients treated with our regimen. It appears that grade 3 febrile neutropenia was more common with our weekly regimen, but that grade 3 neuropathy was more common with the standard regimen. Weekly administration of paclitaxel appears to yield results similar to those of the 3-week schedule administered over a 3-h period with good tolerability.

In the four-arm ECOG trial, for patients who received cisplatin and paclitaxel, overall response rate was 21%, median survival was 7.8 months, and 1-year and 2-year survival rates were 31 and 10%, respectively (Schiller *et al*, 2002).

In the three-arm EORTC trial, for the patients who received cisplatin and paclitaxel, overall response rate was 31.8%, median survival was 8.1 months, and 1-year survival rate was 35.9% (Smit *et al*, 2003). In our regimen, overall response rate was 62.1%, median survival was 13.7 months, and 1-year survival rate was 56.9%. Although the response rate is definitely higher than all of those for the combinations of cisplatin and paclitaxel thus far published, because our phase I/II study was very small in size, there might have been patient selection bias, and seven patients (18.9%) had stage IIIB disease.

In conclusion, we investigated the combination of a weekly paclitaxel and cisplatin regimen. The combination yielded a high response rate, with modest side effects. A phase III study comparing this regimen with 3-week schedule of paclitaxel and cisplatin or carboplatin needs to be performed.
